# Integrating Mendelian randomization and multiple-trait colocalization to uncover cell-specific inflammatory drivers of autoimmune and atopic disease

**DOI:** 10.1093/hmg/ddz155

**Published:** 2019-06-18

**Authors:** Lucy M McGowan, George Davey Smith, Tom R Gaunt, Tom G Richardson

**Affiliations:** 1 School of Physiology, Pharmacology and Neuroscience, Faculty of Life Sciences, University of Bristol, Bristol, BS8 1TD, UK; 2 MRC Integrative Epidemiology Unit, Population Health Sciences Institute, University of Bristol, Bristol, BS8 2BN, UK

## Abstract

Immune-mediated diseases (IMDs) arise when tolerance is lost and chronic inflammation is targeted towards healthy tissues. Despite their growing prevalence, therapies to treat IMDs are lacking. Cytokines and their receptors orchestrate inflammatory responses by regulating elaborate signalling networks across multiple cell types making it challenging to pinpoint therapeutically relevant drivers of IMDs. We developed an analytical framework that integrates Mendelian randomization (MR) and multiple-trait colocalization (moloc) analyses to highlight putative cell-specific drivers of IMDs. MR evaluated causal associations between the levels of 10 circulating cytokines and 9 IMDs within human populations. Subsequently, we undertook moloc analyses to assess whether IMD trait, cytokine protein and corresponding gene expression are driven by a shared causal variant. Moreover, we leveraged gene expression data from three separate cell types (monocytes, neutrophils and T cells) to discern whether associations may be attributed to cell type-specific drivers of disease. MR analyses supported a causal role for IL-18 in inflammatory bowel disease (IBD) (*P* = 1.17 × 10^−4^) and eczema/dermatitis (*P* = 2.81 × 10^−3^), as well as associations between IL-2rα and IL-6R with several other IMDs. Moloc strengthened evidence of a causal association for these results, as well as providing evidence of a monocyte and neutrophil-driven role for IL-18 in IBD pathogenesis. In contrast, IL-2rα and IL-6R associations were found to be T cell specific. Our analytical pipeline can help to elucidate putative molecular pathways in the pathogeneses of IMDs, which could be applied to other disease contexts.

## Introduction

Autoimmune and atopic diseases may arise due to a lack of immune tolerance towards self-antigen or harmless allergens, respectively (1). Loss of immune tolerance results in recurrent or chronic inflammation, causing damage to healthy tissues and extensive morbidity. The incidence of immune-mediated diseases (IMDs) has drastically increased in recent decades, highlighting the need for a clearer understanding of their pathogeneses and effective drug discovery (2). Cytokines and growth factors (herein referred to as cytokines) are signalling factors that orchestrate the balance between immune homeostasis and inflammation via complex signalling pathways (3). However, traditional observational epidemiological studies are prone to confounding and reverse causation, making it challenging to disentangle causal effects of individual cytokines on IMDs (4).

Genome-wide association studies (GWASs) have been instrumental in identifying large numbers of genetic loci that influence disease risk. This includes associations between genes responsible for the synthesis of cytokines and their receptors with autoimmune diseases such as inflammatory bowel disease (IBD) (5–8), multiple sclerosis (MS) (9–11), rheumatoid arthritis (RA) (12) and systemic lupus erythematosus (13), as well as atopic diseases such as eczema (14) and asthma (15). This suggests that particular inflammatory cytokines may have a causal effect on the development of these diseases (4). Previous studies have not yet integrated genome-wide association and cytokine loci data with cell or tissue-specific gene expression loci data to characterize the molecular basis of IMD pathogenesis. Identifying immune cell-specific disease drivers, as well as putative causal relationships between cytokines and IMDs, will help to elucidate complex IMD pathways and identify drug target candidates for therapeutic intervention. Furthermore, targets supported by evidence from statistical analyses of human genetic data are thought to have double the success rate in clinical development (16).

Mendelian randomization (MR) is an increasingly popular statistical method used to strengthen causal inference with respect to exposure–disease associations within human populations, in the absence of confounding variables. MR uses single nucleotide polymorphisms (SNPs), identified through GWAS, as unconfounded proxies for an exposure of interest, analogous to a randomized controlled trial (17). In this study, we have used a conservative (single SNP) and liberal (multiple SNPs) two-sample MR analysis to investigate associations between 11 circulating inflammatory biomarkers (cytokines or cytokine receptors (18–23); Supplementary Material, Table S1) and 9 IMDs (Supplementary Material, Table S2).

We subsequently applied multiple-trait colocalization (moloc) (24) to investigate whether associations detected by MR are due to IMD and circulating cytokine protein sharing a causal variant. Evidence suggesting that they do can strengthen findings from MR that variation in cytokine protein levels has a causal influence on IMD risk. Furthermore, we also integrated data concerning the gene expression of associated cytokines derived from three different cell types (monocytes, neutrophils and T cells) (25). This allowed us to discern whether associations may be due to genetic drivers that influence IMD risk via changes in cell type-specific gene expression. As such, our findings can help develop mechanistic understanding into the regulatory mechanisms underlying associations from GWAS.

## Results

### MR analyses identify putative causal relationships between circulating cytokine/cytokine receptor levels and IMDs

**Figure 1:**
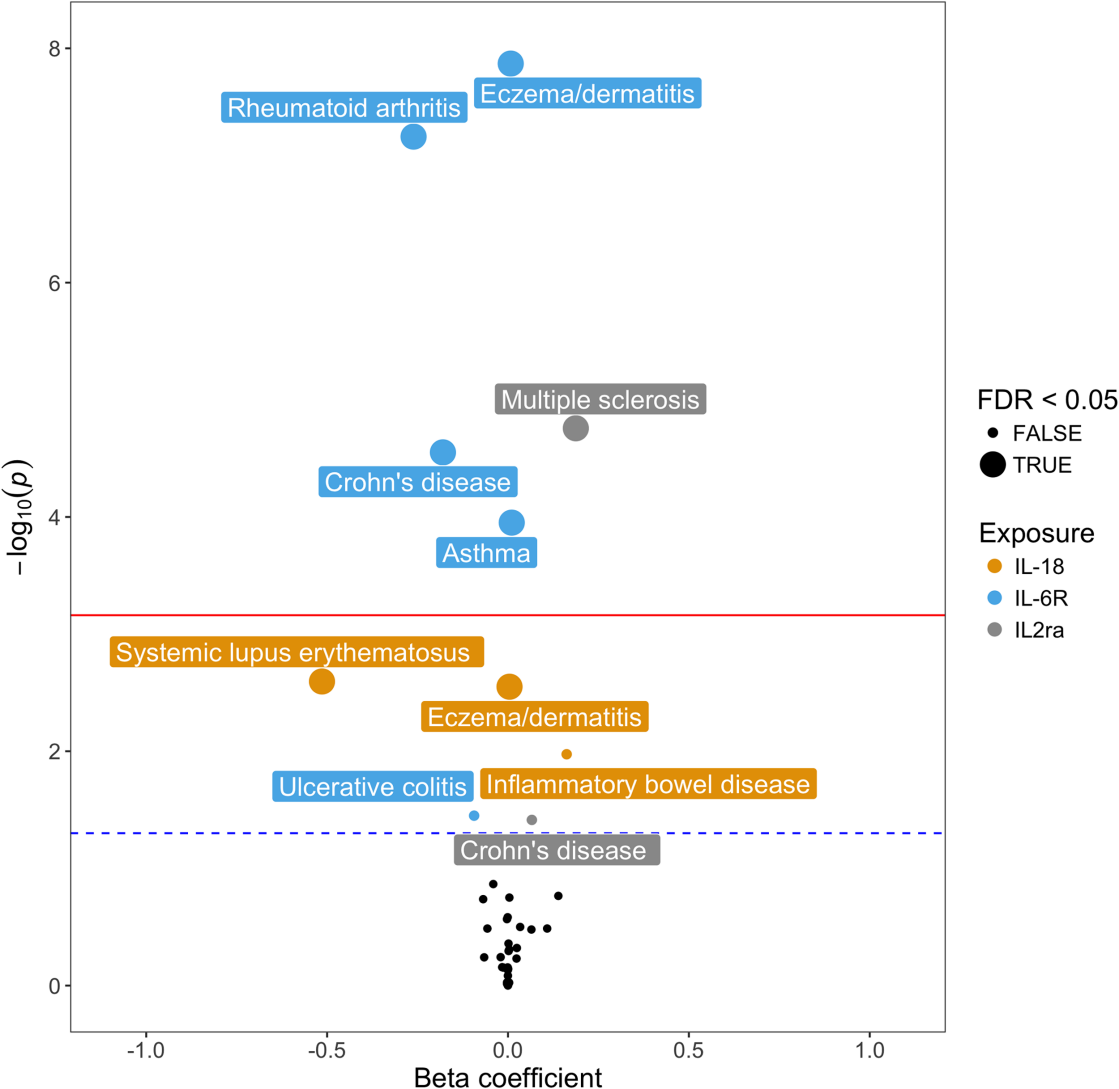
Conservative MR analysis detects associations between circulating inflammatory biomarkers and IMDs. Volcano plot of conservative MR analysis illustrating associations between inflammatory cytokines and complex traits. Red (upper) line represents Bonferroni corrected threshold (*P* ≤ 6.17 × 10^−4^) and black dotted line (bottom) represents unadjusted threshold (*P* ≤ 0.05).

We first used a conservative MR approach to detect associations between inflammatory biomarkers and IMDs, using single *cis*-acting SNPs (i.e. within 1 MB distance of their associated gene) as instruments based on findings from a previous GWAS that reported genetic associations circulating cytokine levels (18). Genes assessed were *IL-18, IL-2Rα, VEGFA, MIF, IL-16, HGF, IL-6R, CRP* and *IL-1R.* We were unable to investigate *IP-10* and *TRAIL* as their *cis*-acting SNPs had minor allele frequencies too rare (<0.05) to undertake formal two-sample MR. Results from the conservative MR analysis are displayed in Fig. 1 and Supplementary Material, Table S3. Based on a Bonferroni corrected *P*-value threshold of *P* ≤ 6.17 × 10^−4^, we identified associations between soluble IL-6R levels and eczema/dermatitis (*P* = 1.35 × 10^−8^), RA (*P* = 5.67 × 10^−8^), Crohn’s disease (*P* = 2.81 × 10^−5^) and asthma (*P* = 1.12 × 10^−4^), as well as an association between IL-2Rα levels and MS (*P* = 1.75 × 10^−5^).

We next performed a liberal MR analysis using all available instruments (i.e. acting in either *cis* or *trans*), associated with our inflammatory biomarkers (Exposure, Supplementary Material, Table S1) as instruments. *IL-6R* (SNPs affecting levels of soluble IL-6R only), *MIF* and *IL-2Rα* were excluded from the liberal analysis as they only had a single *cis*-acting instrument after undertaking linkage disequilibrium (LD) clumping of *r^2^* < 0.001. Our Bonferroni corrected *P*-value threshold for the number of Liberal MR analyses undertaken was *P* ≤ 6.14 × 10^−4^. There was strong evidence of association between circulating levels of IL-18 and IBD (*P* = 1.17 × 10^−4^) and eczema/dermatitis (*P* = 2.81 × 10^−3^). Although these associations were only moderately detected in the conservative analysis using the *IL-18* SNP (rs71478720) alone (*P* = 1.06 × 10^−2^, E/D: *P* = 2.81 × 10^−3^), using multiple instruments in the liberal MR provided stronger evidence of association.

**Figure 2:**
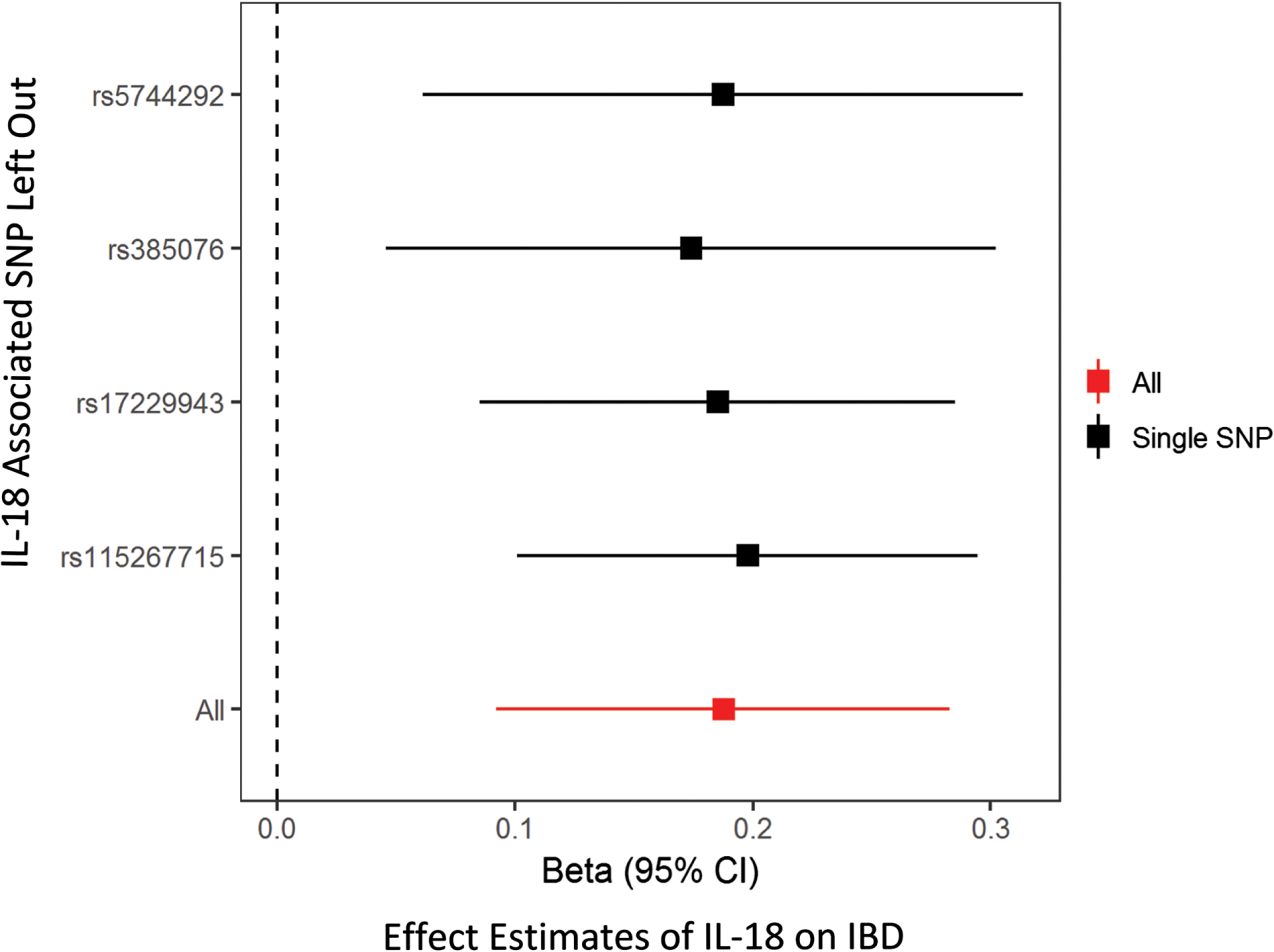
Liberal MR identifies a putative causal relationship between circulating levels of IL-18 and IBD that survives leave-one-out sensitivity analysis. Leave-one-out MR analysis for SNPs used as instruments for liberal MR analysis (black). Results show that this effect is not likely to be due to an individual SNP when compared to the observed effect of all SNPs (red). All SNPs in the leave one out analysis are *trans*-acting.

One of the advantages of the liberal MR analysis is that sensitivity analyses can be performed to test the robustness and the direction of putative inferred causal relationships. Thus, we next conducted a leave-one-out analysis to ensure that no single SNP from the instruments was responsible for the observed effect. Both the analyses of *IL-18* on IBD (Fig. 2) and on eczema/dermatitis (Supplementary Material, Fig. S2) survived leave-one-out analyses (Supplementary Material, Table S5), as the removal of any individual SNP from the analysis had little effect on observed effect estimates. These results provide evidence to support a causal role for circulating IL-18 levels in the pathogenesis of IBD and eczema/dermatitis. Moreover, reverse MR (Supplementary Material, Table S6) and the MR directionality test (26) (Supplementary Material, Table S7) showed that reverse causation was unlikely for any of the associations identified in either the conservative or liberal MR analysis.

### Moloc uncovers immune cell-specific drivers of IMD

For cytokines and IMDs where evidence of a causal association was detected using MR, we applied the moloc method (24) to test whether gene expression for the gene encoding the cytokine, the cytokine protein itself and associated IMD trait all shared the same causal variant. Supplementary Material, Fig. S1B provides an overview of this analysis. At each loci assessed, expression quantitative trait loci (eQTL), protein quantitative trait loci (pQTL) and GWAS effect estimates were used by moloc to assess whether they shared a common causal variant. Furthermore, the moloc analysis was performed 3 times for each association, using eQTL from the BLUEPRINT project derived from either human CD14^+^ CD16^−^ monocytes, CD16^+^ CD66b^+^ neutrophils or CD4^+^ CD45RA^+^ T cells (25).

**Figure 3:**
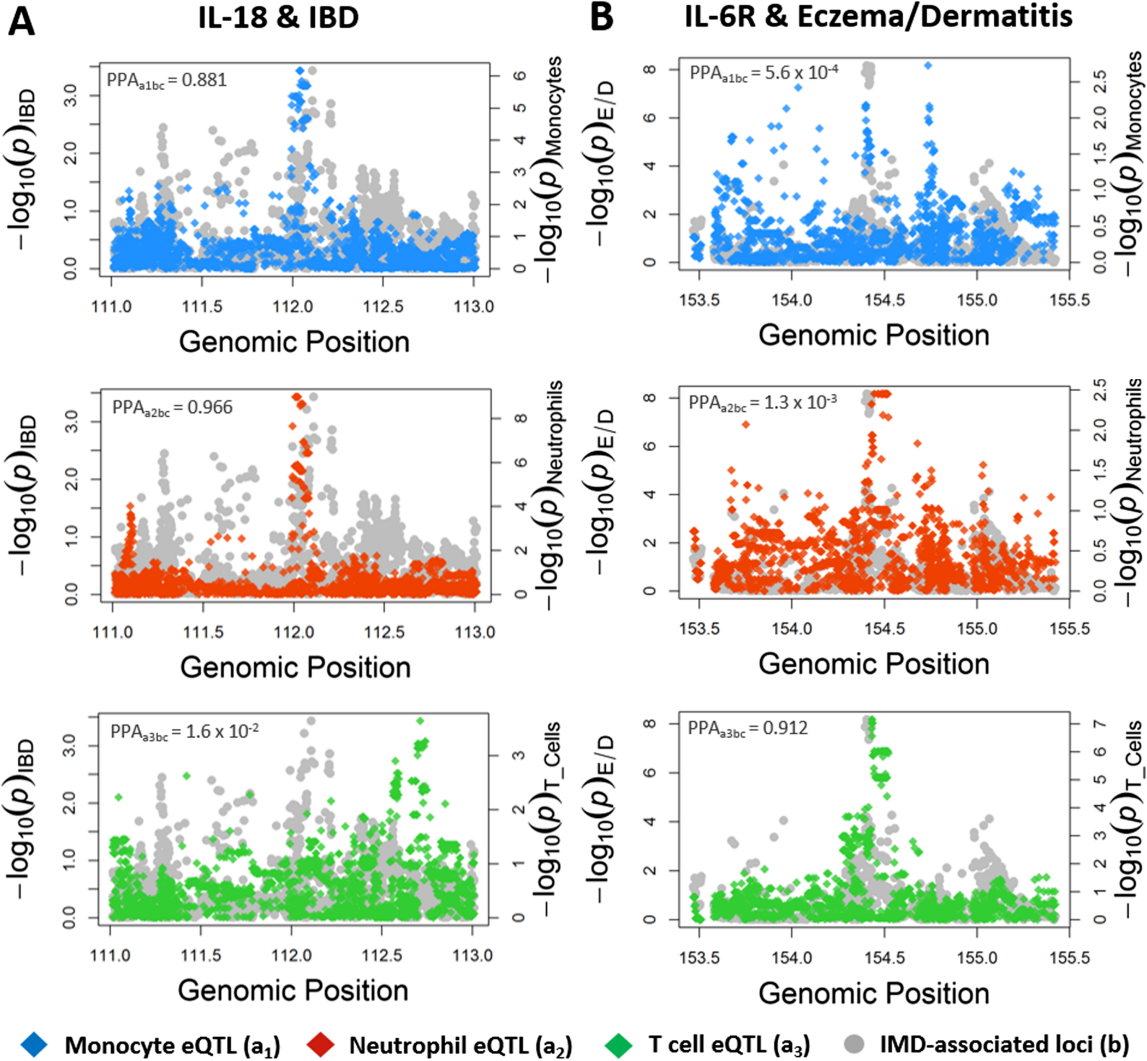
Moloc reveals immune cell-specific divers of IMDs using immune cell eQTL data, inflammatory cytokine or cytokine receptor pQTL data and IMD GWAS data. These plots illustrate observed effects of genetic variants at the *IL-18* (A) and *IL-6R* (B) loci on IBD and eczema/dermatitis (E/D), respectively. Effect estimates on the expression of IL-18 and IL6R are overlaid in each plot using eQTL data derived from monocytes (top, _a1_), neutrophils (middle, _a2_) and T cells (bottom, _a3_). For simplicity, circulating cytokine effects are not displayed within the plots but were used to calculate PPA_abx_ scores. PPA_abc_ values reflect the likelihood that a causal variant influences the target cytokine (_b_), associated complex trait (_c_) and the expression of the corresponding gene (_a_). PPA_abc_ ≥ 0.8 indicates evidence of colocalization (i.e. a shared genetic variant between all three signals) and suggests that the cytokine (or its receptor) is a putative driver of the IMD when it is expressed within the cell type of interest.

We identified evidence of moloc between 10 combinations of immune cell eQTL, IMD-associated loci and inflammatory biomarker pQTL based on a posterior probability of association (PPA) ≥80% (Supplementary Material, Tables S8 and S9). SNPs from each dataset used in the analysis with the strongest *P*-values were reported where evidence of colocalization was detected (Supplementary Material, Table S10). Results indicated that IL-18 plays a role in IBD risk due to changes in gene expression within monocytes (PPA_abc_ = 88.12%) and neutrophils (PPA_abc_ = 96.57%), but not T cells (PPA_abc_ = 1.59%) (Fig. 3A). Additionally, we found evidence of a T cell-specific role in the disease pathways of eczema/dermatitis (PPA_abc_ = 91.15%) driven by soluble IL-6R (Fig. 3B). Likewise, evidence of moloc was identified between IL-2Rα and MS using T cell-derived eQTL data (PPA_abc_ = 88.26%), although this gene was not expressed in monocytes or neutrophils based on findings from the BLUEPRINT study.

We also repeated analyses except using tissue-specific eQTL data derived from the GTEx consortium (27). The association between IL-18 and IBD was relatively ubiquitous, as evidence of colocalization was observed within seven diverse tissue types (Supplementary Material, Table S11), including thyroid tissue (Supplementary Material, Fig. S3a). Evidence of colocalization for the association between soluble IL-6R and eczema/dermatitis was observed in three tissue types, most strongly in whole blood (PPA = 99.52%), which may help shed light on the pleiotropic effects observed at this locus (Supplementary Material, Fig. S3b). Lastly, the association between IL-2rα and MS colocalized in two tissue types: subcutaneous adipose and spleen (Supplementary Material, Fig. S3c). However, other tissue-specific relationships may be masked by the lack of statistical power in current tissue eQTL datasets. As increasing sample sizes of tissue-specific eQTL data emerge, this method could be utilized to test the relationships within a larger number of tissue types.

## Discussion

The ways in which hundreds of functionally diverse cytokines interact and orchestrate inflammatory responses in the pathogenesis of IMDs are unclear (28). In this study, we developed a framework that integrates MR with moloc to gain insights into the molecular basis of IMD pathogenesis. Using this framework, we found evidence to support causal relationships between levels of circulating IL-18 and IBD, as well as eczema/dermatitis, circulating soluble IL-6R and eczema/dermatitis and circulating IL-2Rα and MS, amongst others (Supplementary Material, Tables S3–4, S6–8). Additionally, we provided evidence to suggest that these associations are likely to be driven in an immune cell-specific manner and explored the potential of this method to pinpoint tissue-specific drivers of IMDs.

A T cell-mediated role for IL-2/IL-2Ra in MS has already been well established through epidemiological and lab-based studies (10,29). The IL-2R-targetting drug daclizumab was given FDA (food and drug administration) approval in the United States for the treatment of MS, but was recently withdrawn due to serious side effects (30–32). Additionally, a causal role for IL-18R in atopic dermatitis has recently been described, by integrating MR and pQTL data (33). Our results provide additional evidence to support these existing findings, as well as identifying monocytes and neutrophils as potential drivers of the relationship between IL-18 and eczema/dermatitis (Supplementary Material, Table S8). However, the associations between soluble IL-6R variation and eczema/dermatitis or IL-18 and IBD within human populations are less well understood. Our analyses not only help establish evidence for causal relationships between these inflammatory biomarkers and IMDs but also help characterize their cell type-specific nature.

IL-18, a member of the IL-1 superfamily of cytokines, is a potent inducer of Th1-mediated inflammation and IFN-γ production (34). This cytokine was first linked to IBD nearly 20 years ago, where it was shown to be highly expressed in intestinal tissues derived from IBD patients, compared to healthy control patients (35). Using murine IBD models, deletion of *il-18* or its receptor *il-18r1* has been shown to be protective against inducible colitis, by controlling goblet cell function and maintaining intestinal barrier homeostasis (36). Despite strong evidence to suggest a role for IL-18 in IBD in mice, whether there was a causative role within human populations remained unclear. Our study provides evidence that *IL-18* is likely the causal gene responsible for the association with IBD at this locus, as well as out MR analysis supporting a causal role for IL-18 in the disease pathogenesis of IBD within human populations. Furthermore, the moloc analyses suggested that innate immune cells such as monocytes and neutrophils are likely to drive this association, supporting the current dogma that innate production of IL-18 stimulates Th1/Th17-mediated autoimmunity in IBD (37). The T cell-specific eQTL data currently available for moloc analyses concerned naïve CD4^+^ T cells (38). More eQTL data concerning activated and differentiated immune cell subsets, such as macrophages, dendritic cells and T helper cell subsets (i.e. Th1, Th2, Th17, Treg), are required for additional immune cell subset-specific moloc analysis, to further elucidate the molecular pathways which drive IMDs. Interestingly, small molecule inhibitors that target and repress IL-18-mediated signalling events are currently under development, although not for the treatment of IBD (39). A recently published phase II trial of an IL-18 binding protein (IL-18 bp) drug to treat adult-onset Still’s disease demonstrated a favourable efficacy safety profile (40). If further trails are deemed successful, IL-18 bp drugs may also be used to target other IMDs such as IBD.

IL-6R is the receptor of the pro-inflammatory cytokine IL-6, which can exist in a membrane-bound state on the surface of leukocytes and hepatocytes (classical) or in soluble form (*trans*) (41). Both classical and trans IL-6R signalling culminates in the expression of signal transducer and activator of transcription 3, which promotes inflammation via the expression of genes encoding antiapoptotic proteins and cytokines (42). However, classical IL-6 signalling can act on few cell types compared to trans IL-6 signalling, which can act on any cell that has the cell-bound signal transducer, gp130 (43). One GWAS previously reported an association between elevated soluble IL-6R levels resulting from an SNP in *IL-6R* and atopic dermatitis (44). We provide evidence from the conservative MR and moloc analyses that supports a causal role between soluble IL-6R and eczema/dermatitis. The ratio of classical to trans IL-6R fluctuates as a result of levels cell-bound gp130, receptor shedding by cells expressing membrane-bound IL-6R and changes to the amount of receptor being synthesized by cells (43). Therefore, analyses combining instruments that affect the classical or *trans*-signalling pathways would provide a clearer insight into the role of IL-6 signalling in disease. Interestingly, activated CD4^+^ T cells have been shown to increase levels of soluble IL-6R via IL-6R shedding; this mechanism is thought to have a role in the development of autoimmune diseases, which are often mediated by autoreactive T cells (45,46). Through the moloc analysis, we showed that eczema/dermatitis was likely to be driven by IL-6R expression in T cells (Fig. 3B; Supplementary Material, Table S8). This finding is supported by evidence of increased IL-6R shedding leading to an increase in soluble IL-6R in people diagnosed with atopic dermatitis compared to healthy controls (44).

Relatively few SNPs have been associated with changes in the circulating inflammatory biomarkers investigated in this study, as most of the GWAS summary statistics used to identify our instruments were derived from cohorts with fewer than 9000 people (18,19,23); this is likely due to the high cost of quantification of circulating cytokines and cytokine receptors from blood. Our reverse MR analysis may therefore have been underpowered to evaluate evidence of reverse causation, although supplementing this analysis using the Steiger directionality test also suggested that this was unlikely for the associations we identified. As sample sizes for GWAS of circulating cytokines increase, the analysis pipeline illustrated by this study will have further power to detect novel relationships between markers of inflammation and complex disease. Moreover, our moloc analysis assumes only one causal variant is responsible for associations. While current sample sizes of molecular traits can typically only be instrumented using a single independent *cis*-QTL, future studies uncovering multiple independent QTL on a large scale should benefit from alternative approaches to genetic colocalization. Furthermore, applying MR analyses as proposed in our study in a phenome-wide manner can help elucidate potential adverse side effects of therapeutic intervention (47).

In conclusion, we have found strong evidence supporting new and known causal, immune cell-driven relationships between inflammatory biomarkers and IMDs. Triangulation of results from these analyses, with published results from experimental models of IMDs and GWAS, suggests that targeting IL-18 or its receptor, IL-18R, may be promising for the treatment of IBD. We believe our analysis framework could be applied by other studies with alternative hypotheses, as a way of disentangling complex biochemical cell signalling pathways and identifying molecules and cell types that are likely to drive disease for drug target prioritization.

## Materials and Methods

### Data sources

For our two-sample MR analysis, we harnessed genetic instrument data for 10 circulating inflammatory cytokines or soluble cytokine receptors (Supplementary Material, Table S1) from summary statistics of previously published GWAS (18,19,23,48). The SNP used as an instrument for *IL-6R* affected levels of soluble IL-6R. The SNPs chosen in this analysis have been shown to be robustly associated with a change in circulating levels of a cytokine (*P* < 5 × 10^−8^) and are in *cis* with the gene of interest (i.e. the SNP was located within a 1 MB distance of the gene that encoded the cytokine or cytokine receptor of interest). Data concerning IMD outcomes (Supplementary Material, Table S2) were derived from large-scale GWAS using the MR-Base platform (49). For the moloc analysis, we harnessed human monocyte (CD14^+^ CD16^−^), neutrophil (CD16^+^ CD66b^+^) and T cell (CD4^+^ CD45RA^+^) eQTL data from the BLUEPRINT epigenome project (38). All data used were derived from populations of European descent.

### Mendelian randomization

MR follows three assumptions: (1) that the selected instruments used are robustly associated with the exposure, (2) that the selected instruments are unconfounded and (3) that the selected instruments can only influence the outcome via the exposure. Using randomly inherited unmodifiable SNPs associated with circulating inflammatory cytokine levels through GWAS as genetic instruments for MR satisfies assumptions 1 and 2. We performed two-sample MR with the MR-Base platform (49), using two different analysis methods (Supplementary Material, Fig. S1A) as described below, depending upon available data.

Conservative two-sample MR was used to analyze the causal effect of cytokines using SNPs at target genes encoding the inflammatory biomarkers of interest. As such, for this analysis we used a single SNP acting in *cis* as a genetic instrument (i.e. located within a 1 MB distance of the target gene encoding the cytokine with *P* < 5 × 10^−08^). As only one genetic instrument was used in this analysis, effect estimates were based on the Wald ratio test (50). For single genetic variant *j*, this can be calculated by taking the ratio of the gene–outcome association (denoted by $\hat{\Gamma}$j) and the gene–exposure association (denoted $\hat{\gamma}$j) estimates

$${\hat{\beta}}_j=\frac{\hat{\Gamma} j}{\hat{\gamma j}}.$$

Where possible, liberal MR was also used to analyze the causal effect of circulating inflammatory biomarkers on IMDs. In contrast to the conservative MR analysis, liberal MR used multiple SNPs as genetic instruments that were either acting in *cis* or *trans* (i.e. over 1 MB distance from the target gene encoding the cytokine with *P* < 5 × 10^−08^).

To identify instruments for the liberal MR we undertook genome-wide LD clumping based on *P* < 5 × 10^−08^ and *r^2^* < 0.001. A leave-one-out MR analysis was performed in parallel with liberal MR to ensure that causal effects were not observed due to the influence of a single SNP. As two or more genetic instruments were available for liberal MR, we used the inverse variance weighted method to obtain MR effect estimates (50).

$${\hat{\beta}}_{IVW}=\frac{\sum_j{\hat{\gamma}}_j^2{\sigma}_{Yj}^{-2}{\hat{\beta}}_j}{\sum_j{\hat{\gamma}}_j^2{\sigma}_{Yj}^{-2}},$$

where ${\sigma}_{Yj}$ is the standard error of the gene-outcome estimate for variant *j*.

For both conservative and liberal MR analyses, we used the Bonferroni correction to calculate an adjusted *P*-value threshold (conservative: *P* ≤ 6.17 × 10^−4^, liberal: *P* ≤ 6.94 × 10^−4^) to account for multiple testing, where

$$Threshold=\frac{0.05}{No. Exposures\ \mathrm{X}\ No. Outcomes}.$$

We subsequently undertook reverse MR analyses for all associations that survived multiple testing to investigate reverse causation (i.e. the likelihood that genetic predisposition to disease has an influence on inflammatory cytokine levels). As an additional sensitivity analysis, we used the MR Steiger directionality test to assess the directionality of associations between inflammatory cytokines and complex traits (26).

### Multiple-trait colocalization analysis

We applied the moloc approach (24) to immune cell-specific eQTL data, inflammatory biomarker pQTL data and IMD-associated loci data to identify putative immune cell-specific drivers of IMDs (Supplementary Material, Fig. S1B). The moloc method uses a Bayesian statistical framework to calculate PPA scores to measure the degree of colocalization between gene loci using three or more datasets. PPA scores of ≥80% are likely to share a common genetic causal variant based on evaluations undertaken by the authors (24). We obtained eQTL data derived from concerning neutrophils (CD16^+^ CD66b^+^), monocytes (CD14^+^ CD16^−^) and T cells (CD4^+^ CD45RA^+^) (38). We ran independent analyses for each of the three immune cell types, testing the degree of colocalization between immune cell eQTL, with inflammatory biomarker pQTL and IMD-associated loci.

We chose to perform moloc for inflammatory biomarkers and IMDs in this instance based upon a *P*-value threshold <0.05 in either the conservative or liberal MR analysis. Along with investigating cell-type specificity for identified associations, this analysis was used to detect evidence of a coordinated system that is consistent with causality (i.e. gene expression and respective protein products colocalize with the associated complex traits). As such these findings can complement evidence from MR to detect putative causal effects between biomarkers and disease.

Finally, we undertook exploratory moloc analyses to investigate gene expression within different tissue types using data from the GTEx consortium v6p (51). Our three traits in each analysis were the circulating inflammatory cytokine using pQTL data, the complex trait with strongest evidence of association with the cytokine in either the liberal or conservative MR analyses and tissue-specific gene expression for the cytokine using GTEx eQTL data. We only investigated tissue types with at least one eQTL (*P* < 1.0 × 10^−04^) for the target cytokine gene due to the small sample sizes concerning tissue-specific eQTL in the GTEx database. As in the immune cell eQTL moloc analysis, evidence of colocalization was based on a PPA score of ≥80%.

All statistical and bioinformatics analyses were undertaken using R statistical software version 3.31 (52). Plots illustrating multiple trait colocalization were generated using base R graphics, whereas our volcano plot was generated using ggplot (53).

## Supplementary Material

Supplementary_figures_REVISED_ddz155

Supplementary_tables_TGR_ddz155
