# Supplementary material for: Integrating Mendelian randomization and multiple-trait colocalization to uncover cell-specific inflammatory drivers of autoimmune and atopic disease
Source: Hum Mol Genet. 2019 Jun 18;28(19):3293–300. doi: 10.1093/hmg/ddz155 (PMC6859431; doi:10.1093/hmg/ddz155)
Supplement: Supplementary_figures_REVISED_ddz155 [file supplementary_figures_revised_ddz155.pdf]

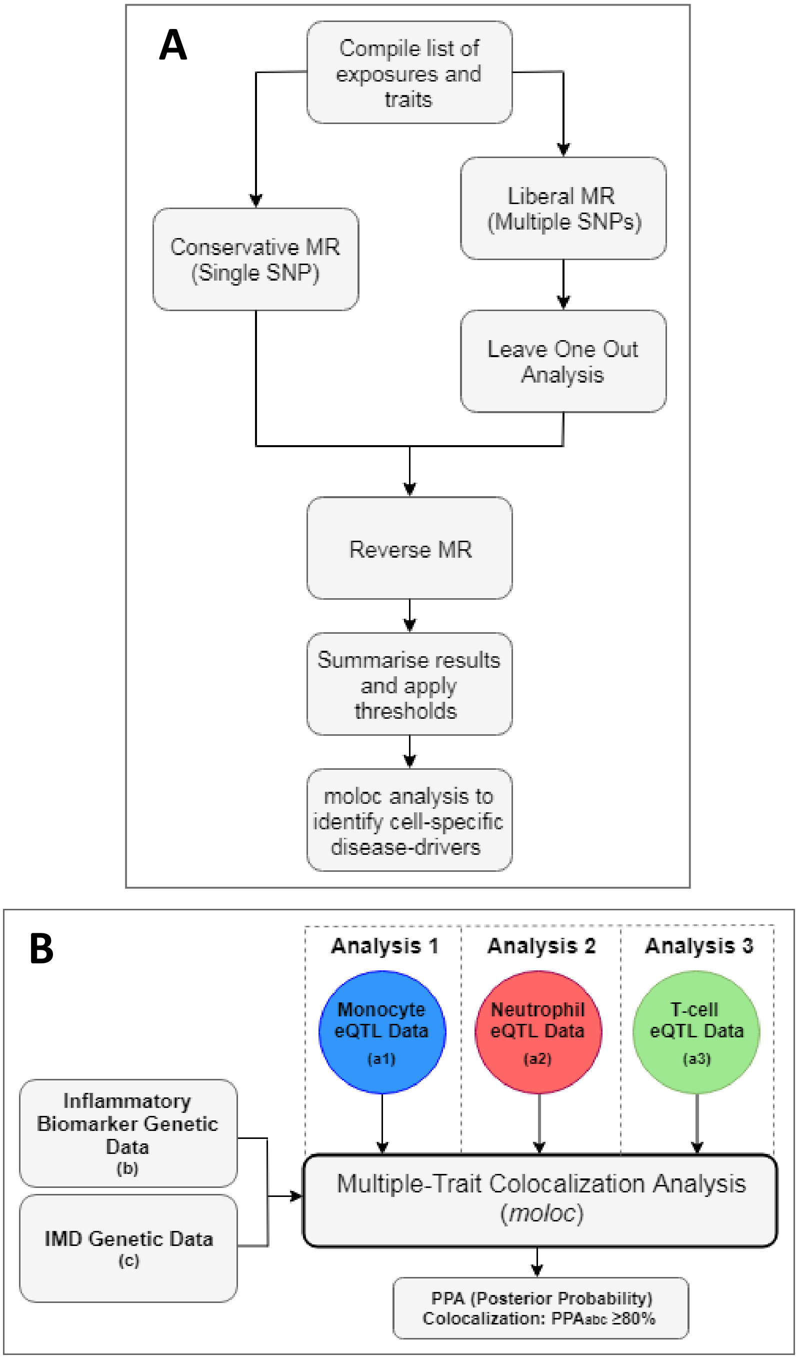

Figure S1. Schematics of Mendelian Randomization (MR) and multiple trait colocalization (moloc) analyses performed in this study. A: Flowchart outlining MR analyses protocol used to assess associations between circulating inflammatory biomarker levels (exposures) and IMDs (traits). Each analysis was performed depending upon data availability and suitability for the method. Moloc was only performed to identify likely immune cell-specific drivers of IMDs where a at least one causal relationship (below P value threshold) was identified between an inflammatory biomarker and IMD using MR B: Schematic of moloc method where the degree of colocalization between expression quantitative trait loci (eQTL) from 3 immune cell-types (a_1 = monocytes, a_2 = neutrophils and a_3 = CD4$^+$ T cells) were measure individually against inflammatory biomarker-associated genetic loci (b) and IMD-associated genetic loci (c).

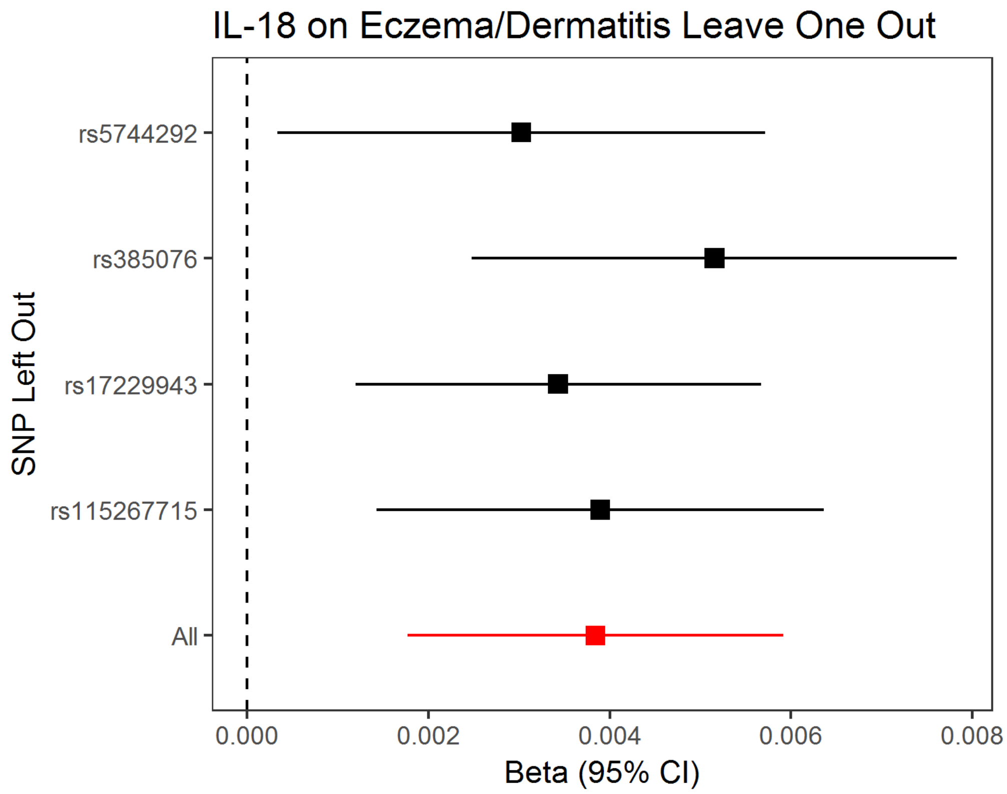

Figure S2. Forest plot from leave one out MR analysis of IL-18 on eczema/dermatitis. Liberal two-sample MR supports a causal role for IL-18 in eczema/dermatitis pathogenesis which survives leave one out analysis. All SNPs used in the leave one out analysis are *trans*-acting.

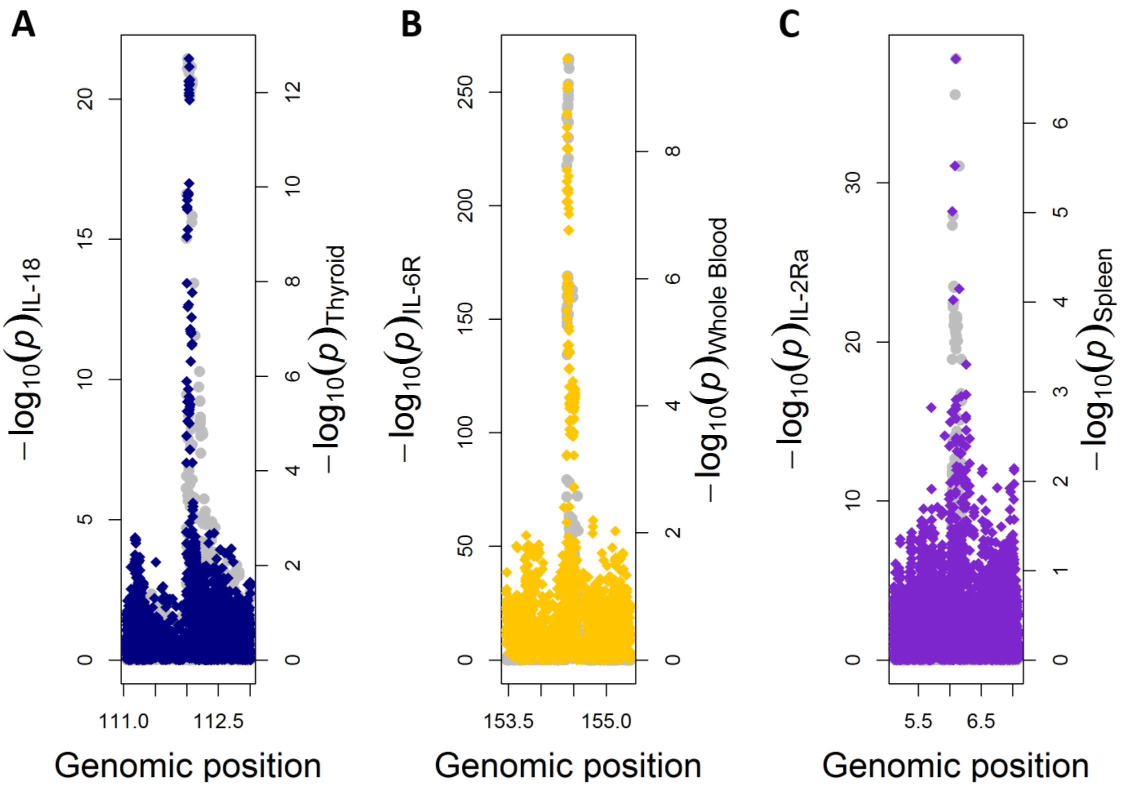

Figure S3. Multiple-trait colocalization indicates tissue specific expression of genes which are associated with circulating cytokine or cytokine receptor levels and immune-mediated disease (IMD) susceptibility. These plots illustrate observed effects of genetic variants at the *IL-18* (A), and *IL-6R* (B) and *IL-2Rα* (C) loci with their corresponding protein product. Effect estimates on the expression of IL-18, IL-6R, IL-2Rα are overlaid in each plot using expression quantitative trait loci data derived from thyroid (A), whole blood (B) and spleen (C). For simplicity, effects on complex traits are not displayed within the plots but were used to calculate PPA_{abx} scores. PPA_{abc} values reflect the likelihood that a causal variant influences the target cytokine ($_b$), associated complex trait ($_c$) and the expression of the corresponding gene ($_a$). $PPA_{abc} \geq 0.8$ indicates evidence of colocalization (i.e. a shared genetic variant between all 3 signals) and suggests that the cytokine (or its receptor) is a putative driver of the IMD when it is expressed within the tissue-type of interest.
